# Characterization of Pax3 and Sox10 transgenic *Xenopus laevis* embryos as tools to study neural crest development

**DOI:** 10.1016/j.ydbio.2018.02.020

**Published:** 2018-12-01

**Authors:** Mansour Alkobtawi, Heather Ray, Elias H Barriga, Mauricio Moreno, Ryan Kerney, Anne-Helene Monsoro-Burq, Jean-Pierre Saint-Jeannet, Roberto Mayor

**Affiliations:** a UMR3347 Université Paris Sud-Paris Saclay, Institut Curie/CNRS/U1021 INSERM, Centre Universitaire bât, 110 91405 ORSAY Cedex, Paris, France; b Dept. of Cell, Developmental and Integrative Biology, The University of Alabama at Birmingham MCLM 338, 1918 University Dr. Birmingham, AL 35294, USA; c Department of Cell and Developmental Biology, University College London, Gower Street, WC1E 6BT, London, UK; d Department of Biology, Gettysburg College Gettysburg, PA 17325, USA; e Institut Universitaire de France, 75005, Paris France; f New York University, College of Dentistry, Department of Basic Science&Craniofacial Biology, New York, NY 10010, USA

**Keywords:** Neural crest, transgenic, Pax3, Sox10, induction, migration

## Abstract

The neural crest is a multipotent population of cells that originates a variety of cell types. Many animal models are used to study neural crest induction, migration and differentiation, with amphibians and birds being the most widely used systems. A major technological advance to study neural crest development in mouse, chick and zebrafish has been the generation of transgenic animals in which neural crest specific enhancers/promoters drive the expression of either fluorescent proteins for use as lineage tracers, or modified genes for use in functional studies. Unfortunately, no such transgenic animals currently exist for the amphibians *Xenopus laevis* and *tropicalis,* key model systems for studying neural crest development. Here we describe the generation and characterization of two transgenic *Xenopus laevis* lines, Pax3-GFP and Sox10-GFP, in which GFP is expressed in the pre-migratory and migratory neural crest, respectively. We show that Pax3-GFP could be a powerful tool to study neural crest induction, whereas Sox10-GFP could be used in the study of neural crest migration in living embryos.

## Highlights

• Pax3-GFP Xenopus laves transgenic expresses GFP in pre-migratory neural crest
• Pax3-GFP Xenopus laevis transgenic responds to Wnt signalling
• Sox10-GFP Xenopus laevis transgenic expresses GFP in migrating neural crest
• Pax3-GFP and Sox10-GFP Xenopus laevis transgenic represent potential tools to study neural crest induction and migration

## 1 Introduction

The neural crest is a multipotent population of cells that arises at the border between the developing nervous system and surface ectoderm and originates a variety of cell types, which contribute to the formation of many organs and systems (LaBonne and Bronner-Fraser, 1999, Huang and Saint-Jeannet, 2004, Bronner and Simões-Costa, 2016, Mayor and Theveneau, 2013). This multipotent stem cell population has fascinated biologists for more than a hundred years, as it represents an ideal system to study embryonic induction, epithelial-to-mesenchymal transition, cell migration, differentiation, and evolution. The multipotency of the neural crest renders it of great interest for regenerative medicine and as a therapeutic target for syndromes associated with this cell population, including many craniofacial defects (Trainor and Andrews, 2013).

Neural crest cells are a vertebrate invention (Gans and Northcutt, 1983) with potential homologous cell populations in basal chordates. Many animal systems have been used to study the neural crest (Barriga et al., 2015). Amphibian and birds have been the most widely used animals to study induction, migration and differentiation of neural crest cells; and more recently mouse and teleosts have joined this list. Evolutionary studies have been done in basal vertebrates such as lamprey and hagfish (Sauka-Spengler et al., 2007, Ota et al., 2007).

Chick, frog, mouse and zebrafish are the animal models that have most contributed to our understanding of neural crest development. In particular, many of the signals involved in neural crest induction and the mechanisms that control neural crest migration were identified in chick and *Xenopus* (Milet and Monsoro-Burq, 2012; Theveneau and Mayor, 2012), and the advent of molecular genetics has revolutionized the field (Bronner and Simões-Costa, 2016). A major technological advance to study neural crest development has been the generation of transgenic animals in which neural crest specific enhancers/promoters drive the expression of reporter genes in the neural crest. The use of genetically encoded labelling of neural crest cells has allowed the purification of neural crest subpopulations in chick (Simoes-Costa and Bronner, 2016), and the study of neural crest migration in live zebrafish and mouse embryos (Rodrigues et al., 2012, Ray and Niswnader, 2016, Corpening et al., 2011, Shibata et al., 2010). Unfortunately, *Xenopus spp*., which are ideally suited for exploring early NC formation, lag behind in the availability of these powerful transgenic tools to study neural crest development.

Here we describe the generation and characterization of two transgenic *Xenopus laevis* lines, Pax3-GFP and Sox10-GFP, with potential for use in neural crest induction and migration studies, respectively. We show that Pax3-GFP transgenic embryos express GFP in early neural crest cells and respond to neural crest inductive signals, and that Sox10-GFP transgenic embryos express GFP in migrating neural crest, both fluorescent labels which are readily visible in living embryos.

## 2 Material and Methods

### 2.1 Cloning and transgene generation

A 2.9 kb HindIII-EcoR1 fragment located upstream of the *pax3* coding region was cloned into SalI-XbaI digested Tol2 vector T2AL200R150G (Suster et al., 2009) to form Pax3-GFP. Transient transgenic embryos were generated by injecting 200 pg of the Pax3-GFP DNA construct dissolved in 8 nl of water into both blastomeres of a 2-cell stage embryo as previously described (Mayor et al., 1993). Stable transgenic animals for Pax3 were generated as described in Loeber et al. (2009). For the Sox10-GFP construct, a 5.1 kb sequence was cloned with 5’ Sal1 and 3’ Nsi1 sites using the PCR primers Sox10FSal1 (GTCGACAAGTCGACCATGAGCCTGGCCTA) and Sox10RNsiI (ATGCATAAATGCATTTCCAAGAGCGATGTGATTGG), into a reporter vector that has a separate gamma crystallin - GFP reporter as an internal positive control for the transgenics. The amplicon was sub-cloned into the ISce1 vector with HS4 insulators as described in Rankin et al. (2009). The generation stable transgenic frogs for Sox10 was performed as described in Ogino et al. (2006a).

### 2.2 Fertilization, embryo generation and collection

Wild-type female *Xenopus laevis* were obtained from the National Xenopus Resource (NXR, Woods Hole, Mass) and housed at the Cold Spring Harbor Laboratory for use in the Cell and Developmental Biology of *Xenopus* course. Female frogs were injected with 800U of human chorionic gonadotropin and mature oocytes were collected 12–16 hours later. For the generation of Sox10-GFP embryos, eggs were *in vitro* fertilized with sperm from transgenic male *X.l.* frogs that express green fluorescent protein (GFP) under control of the endogenous Sox10 promoter (Xla.Tg(sox10:GFP)^Jpsj^, provided by NXR). For the generation of Pax3-GFP embryos, oocytes were fertilized with sperm from transgenic male frogs that express GFP under the Pax3 promoter (Xla.Tg(2.9pax3:GFP)EXRC). Fertilized embryos were allowed to develop until the appropriate stage for live imaging or fixation in 4% PFA for one hour at room temperature. All animal use was conducted in compliance with the Institutional Animal Care and Use Committee at the Cold Spring Harbor Laboratory or according to instructions from the Home Office of the United Kingdom, where animal licenses are required. Embryos were obtained and staged as described previously (Nieuwkoop and Faber, 1967). Transgenic lines generated in this study (Pax3:GFP and Sox10:GFP) are maintained at the European Xenopus Resource Centre (EXRC) and Xenopus National Resource (XNR, Sox-10:GFP only) and available upon request.

### 2.3 Whole-mount in situ hybridization (WISH)

Embryos were fixed in 4% Formaldehyde for 2 hours and dehydrated in 100% Ethanol. Whole-mount in situ hybridization was performed according to an optimized protocol for superficial structures (see for more details Monsoro-Burq, 2007). Anti-sense digoxygenin-labelled *pax3* and *sox10* probes (Bang et al., 1997, Aoki et al., 2003) were synthesized *in vitro* and used at a final concentration of 1 μg/1 ml. Embryos were post-fixed, bleached and images were taken using a Lumar V12 Binocular microscope equipped with bright field and color camera (Zeiss).

### 2.4 Imaging the expression of Pax3-GFP and Sox10-GFP and time lapse microscopy of Sox10-GFP embryos

Pictures of live embryos and larvae stably expressing Pax3-GFP or Sox10-GFP were taken using a Leica M205 FA Stereo fluorescence microscope. For time-lapse imaging, Stage 21 *Xenopus laevis* embryos expressing Sox10-GFP were placed in 1/3 MMR medium and images were taken every 6 minutes for 12 hours using a Leica M205 FA Stereo fluorescence microscope.

### 2.5 Immunofluorescence and cryosection

After fixation, embryos were washed in phosphate buffered saline (PBS), dehydrated through two changes of 100% ethanol and two changes of 100% methanol (15 minutes each), and stored at −20° C in methanol. Embryos were rehydrated into PBS prior to 48-hour incubation in 20% sucrose in PBS at 4 ° C. Embryos were then equilibrated into OCT compound (Fisher Scientific, Waltham, MA) through graded washes of 25:75, 50:50, 75:25, 100:0 OCT:20% sucrose (performed at room temperature, 30 min. each). Single embryos were embedded in OCT, snap frozen on dry ice, and stored at −80° C. 12μm transverse embryo sections were obtained through cryo-sectioning and allowed to dry on glass slides at room temperature overnight. For immunostaining, sections were rehydrated in PBS (3 × 5 min.), permeabilized with 0.1% Tween-20 in PBS (1 × 10 min.), incubated in permeabilization buffer with 10% normal goat serum (block buffer, 1 × 1 hour), followed by overnight incubation with primary antibody at 4 ° C (rabbit anti-GFP [Sigma Aldrich, St Louis, MO] at 1:200 dilution in block buffer). Sections were then washed with PBS (3 × 5 min.) and incubated with secondary antibody for 2 hours at room temperature (Goat anti-rabbit AF 555 [Life Technologies, Carlsbad, CA] at 1:1000 dilution in permeabilization buffer). After final washes (PBS, 3 × 15 min.), slides were mounted with Fluoromount-G with DAPI (eBioscience, San Diego, CA). Imaging was performed using an Olympus FV1000 laser scanning confocal microscope with a 10× objective, and images were processed using ImageJ to present maximum projections of multiple z-planes.

### 2.6 Dexamethasone treatment

Dexamethasone treatment was performed as described by Kolm and Sive (1995). Dexamethasone was included in the culture medium at the indicated stages and maintained until the embryos were fixed.

## 3 Results and Discussion

### 3.1 Generation of Pax3-GFP transgenic embryos

**Fig. 1:**
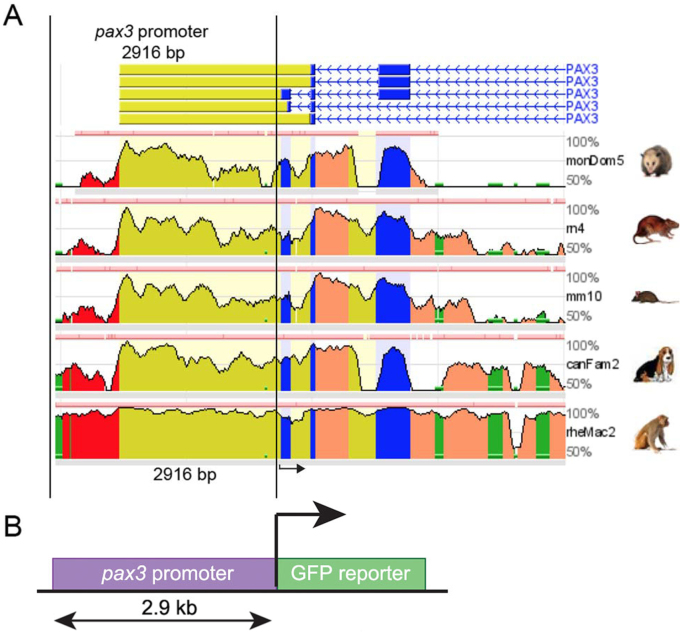
**Characterization of a*****pax3*****promoter.** (A) Graphical representation showing the result of a comparative genomic analysis obtained using the ECR browser. The genomic region corresponding to the vertebrate *pax3* gene was compared among: opossum, rat, mouse, dog, and macaque. The *pax3* promoter region (2916 bp) is delimited by two black parallel lines. Black arrow indicates the initiation site. bp, base-pairs. (B) Schematic showing a simplified view of the transgene containing the *pax3* promoter (2916 bp) driving expression of the green-fluorescent-protein (GFP). Key: red, highly conserved elements; blue, coding exons; yellow, UTRs; green, transposable elements and simple repeats.

To determine the regulatory regions of *pax3* in *Xenopus laevis*, comparative genomic analysis was employed to identify conserved elements. Genomic sequences surrounding the *pax3* coding region from different vertebrate species were compared *in silico* (Fig. 1A), using the Vista ECR browser program. Using a *pax3* BAC clone, a genomic fragment of ∼3 kb, containing ≥29.6% of homology with the vertebrate species analyzed here, was cloned into a GFP reporter vector upstream of a thymidine kinase (tk) basal promoter (Uchikawa et al., 2003). The GFP sequence was placed so that the ATG sequence of GFP is in the same relative position as the *pax3* ATG (Fig. 1B). The construct was then used to test if this region contains sufficient regulatory elements to recapitulate Pax3 expression during early neural crest formation.

**Fig. 2:**
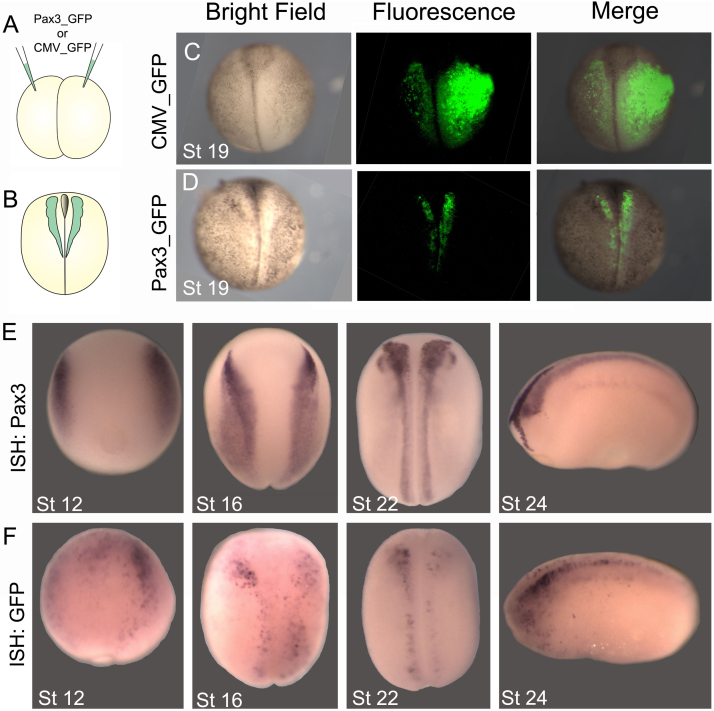
**Generation of Pax3-GFP transient transgenic embryos.** (A) *pax3* or *CMV* promoters fused to GFP were injected into *Xenopus* embryos as described to generate transient transgenic embryos. (B) GFP fluorescence was examined at neurula stage (stage 19). (C) CMV-GFP transient transgenic embryo, showing ubiquitous expression. (D) Pax3-GFP transient transgenic embryo showing expression restricted to the neural folds. (E, F) In situ hybridization (ISH) for comparison of endogenous Pax3 expression with GFP expression in Pax3-GFP transient transgenic embryos. (E) ISH for *pax3* at the indicated stages. (F) ISH for GFP in Pax3-GFP embryos at the indicated stages. Note the similar expression of Pax3 and GFP. 92% of Pax3-GFP embryos exhibited expression in the neural folds (n= 210).

Pax3-GFP and CMV-GFP (control) plasmids were injected into both blastomeres of two-cell stage embryos (Fig. 2A) as described in the Materials and Methods to generate transient transgenic embryos, and GFP fluorescence was analyzed at neurula stage (Fig. 2B). Injection of the CMV-GFP construct results in ubiquitous expression (Fig. 2C) as expected for the CMV promoter, whereas GFP expression upon injection of the Pax3-GFP construct is restricted to the neural folds within the expected Pax3 expression domain (Fig. 2D). To compare GFP expression driven by the Pax3-GFP construct with endogenous Pax3 expression, *in situ* hybridization for Pax3 in wild type embryos and for GFP in Pax3-GFP transient transgenic embryos were compared. Pax3 is normally expressed at the border between the neural and non-neural ectoderm (stage 12), in the prospective neural folds of early neurula embryos (stage 14), and later becomes localized within the neural folds to include the prospective neural crest and hatching gland (stage 16) (Monsoro-Burq et al., 2005). Pax3 expression remains in the neural tube after neurulation is completed, but is switched off in the migrating neural crest, and persists in the superficial hatching gland cells (Fig. 2E; Bang et al., 1997; Monsoro-Burq et al., 2005; Hong and Saint-Jeannet, 2007). Plasmids injected into *Xenopus* embryos often do not express as well as endogenous genes, probably due to limited diffusion of the plasmid and subsequent unequal inheritance by daughter cells. This explains the spotty pattern of expression in the Pax3-GFP transient transgenic embryos as compared with the endogenous Pax3 expression (Fig. 2E, F). Despite this limitation, a similar overall pattern of GFP expression is observed in the neural crest of Pax3-GFP transient transgenic embryos but not in the hatching gland (Fig. 2F), indicating that the DNA sequence upstream of Pax3 used to generate the transgenic contains all the regulatory elements required to recapitulate Pax3 expression in neural crest progenitors and pre-migratory neural crest.

### 3.2 Wnt signaling response of Pax3-GFP transgene

**Fig. 3:**
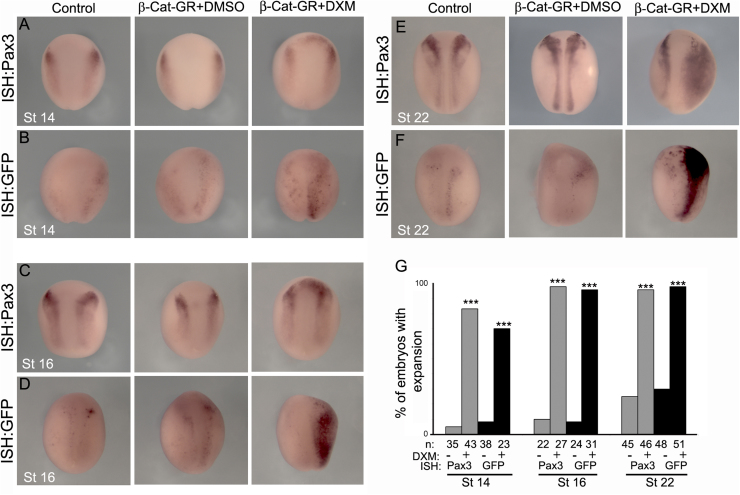
**Pax3-GFP responds to Wnt signaling.** (A, C, E) ISH for endogenous Pax3 in wild type embryos at the indicated stages. (B, D, F) ISH for GFP in Pax3-GFP transient transgenic embryos at the indicated stages. Embryos were non-injected (Control) or injected with an inducible form of β-catenin (β-Cat-GR) and treated with DXM (in DMSO) to induce β-catenin, or DMSO alone. (G) Quantification of the percentage of embryos that showed expansion in Pax3/GFP expression. Note that activation of β-catenin leads to an equivalent increase of Pax3 and GFP expression. *** P<0.001.

As it has been shown that Pax3 expression is positively regulated by Wnt signaling at the neural/non-neural ectoderm border (NB), which is among the most important early NB-NC inducing signals (Monsoro-Burq et al., 2005, Sato et al., 2005, Hong and Saint-Jeannet, 2007), we tested whether the Pax3-GFP construct was able to respond to Wnt signaling in transient transgenic *Xenopus* embryos. Two-cell stage embryos were injected into the right side blastomeres with an inducible form of β-catenin (β-Cat-GR) that is activated upon dexamethasone addition (Domingos et al., 2001). Wild-type embryos activated at stage 11 and fixed at stages 14, 16 and 22 (Nieuwkoop and Faber, 1967) show a clear induction of Pax3 expression upon dexamethasone treatment as compared with DMSO control embryos (Fig. 3A,C, E, quantification in Fig. 3G). An equivalent upregulation of GFP is observed in Pax3-GFP transient transgenic embryos (Fig. 3B,F,D,G), indicating that the Pax3-GFP transgene responds to Wnt signaling in a similar manner as the endogenous *pax3* gene.

These results show that the *pax3* 2916 bp genomic fragment contains sufficient elements to respond to neural crest inductive signals. Thus, this genomic region could potentially be used to study the molecular aspects of neural crest induction by identifying the transcription factors that bind and regulate the Pax3 promoter. In addition, this fragment could be cloned upstream of any gene to drive neural crest-specific protein expression. Although the data presented here is from Pax3-GFP transient transgenic embryos, similar patterns of expression are observed in stable Pax3-GFP transgenic frogs. Stable transgenic lines have been generated and are maintained at the European Xenopus Resource Centre (EXRC) where they are available upon request. It is important to note that, like endogenous Pax3 expression, the Pax3-GFP transgene is switched off as soon as neural crest migration starts, and therefore this transgenic *Xenopus* line cannot be used to study neural crest migration.

### 3.3 Generation and characterization of Sox10-GFP transgenic embryos

**Fig. 4:**
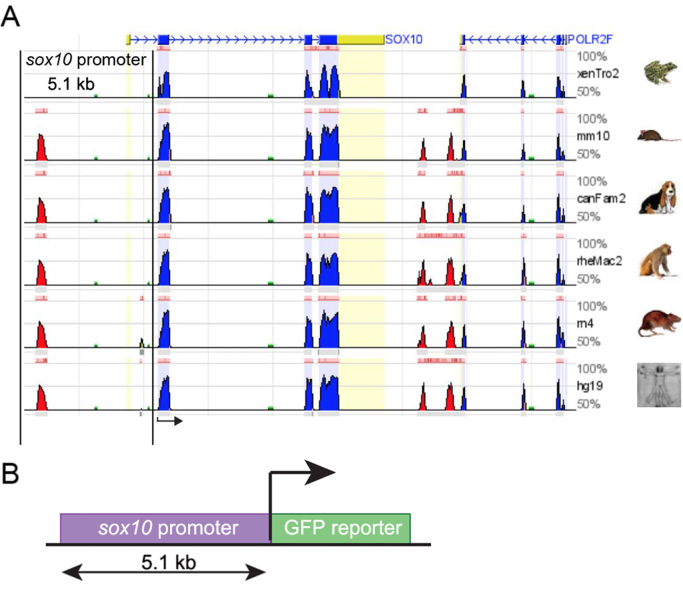
**Characterization of*****sox10*****promoter.** (A) Graphical representation showing the result of a comparative genomic analysis obtained using the ECR browser. The genomic region corresponding to the vertebrate *sox10* gene was compared among: *Xenopus tropicalis*, mouse, dog, macaque, rat, and human. The *sox10* promoter region (5.1 kb) is delimited by two black parallel lines. (B) Schematic showing a simplified view of the transgene containing the *sox10* promoter (5.1 kb) driving the expression of the green-fluorescent-protein (GFP). Key: red, highly conserved elements; blue, coding exons; yellow, UTRs; green, transposable elements and simple repeats. Black arrow indicates the initiation site. bp, base-pairs.

As the Pax3-GFP transgene is not expressed in migrating neural crest, we decided to generate a Sox10-GFP *Xenopus laevis* transgenic frog. The *sox10* gene was selected due to its strong expression during neural crest migration (Aoki et al., 2003, Honore et al., 2003). In order to identify a neural crest-specific regulatory element of the *Xenopus laevis sox10* gene, an *in silico* analysis similar to the one described for *pax3* was performed. Genomic sequences upstream of the *sox10* coding region from various vertebrate species were compared *in silico* (Fig. 4A), using the Vista ECR browser. A 5.1 kb fragment was identified and cloned into a GFP reporter vector upstream of a thymidine kinase (tk) basal promoter (Uchikawa et al., 2003) as described for the Pax3-GFP construct (Fig. 4B). Injection of the Sox10-GFP plasmid into *Xenopus* embryos generated a pattern of GFP expression similar to endogenous Sox10 expression indicating that the major Sox10 regulatory elements are present in the 5.1 kb fragment. Stable transgenic *Xenopus laevis* animals were generated with the 5.1 kb fragment as described in the Materials and Methods. As the main aim for generating this transgenic *Xenopus laevis* was to label the migrating neural crest, and because the expression of GFP in the Sox10-GFP is weak in the pre-migratory neural crest (data not shown), we focus our description of this new transgenic line on the neural crest migratory stages, where the expression of Sox10-GFP is stronger.

**Fig. 5:**
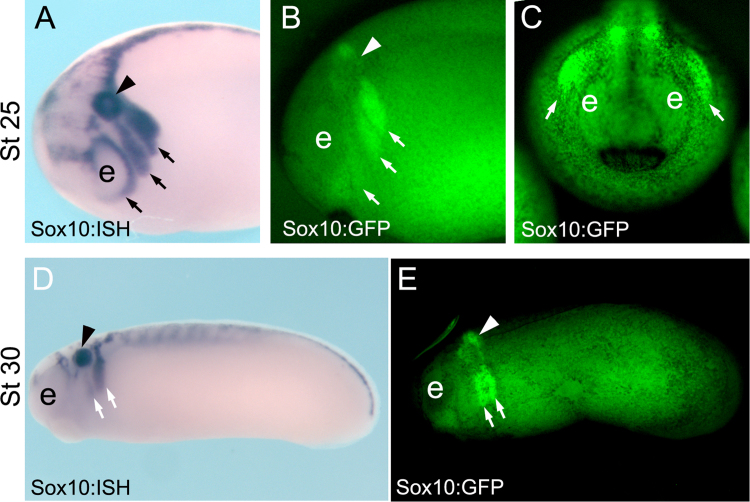
**Characterization of Sox10-GFP stable transgenic embryos.** (A-C) Stage 25 embryos. (A) ISH for endogenous *sox10* in wild-type embryos at the indicated stage, side view. (B, C) GFP fluorescence of Sox10-GFP stable transgenic embryos at the indicated stage, B, side view and C, anterior view. (D, E) Stage 30 embryos, side view. (D) ISH for endogenous *sox10* in wild type embryos at the indicated stage. (E) GFP fluorescence of Sox10-GFP stable transgenic embryos at the indicated stage. Note the similar expression of Sox10 and GFP in the migrating neural crest cells (arrows). Arrow head indicates the otic vesicle and e the eye.

**Fig. 6:**
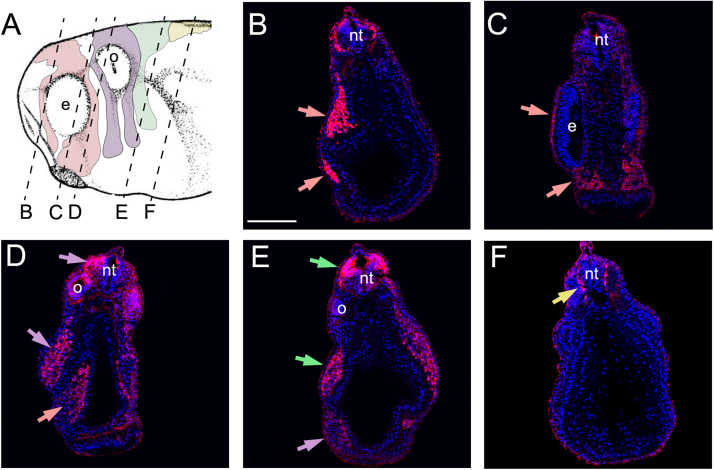
**GFP expression in migrating neural crest in Sox10-GFP stable transgenic embryos.** (A) Diagram indicating the level of the transverse sections shown in panels B-F. Pink shows mandibular neural crest; Purple shows hyoid neural crest; Green shows branchial neural crest; Yellow shows trunk/vagal neural crest. (B-F) Sections of Sox10-GFP stable transgenic embryos after immunostaining for GFP and DAPI. (B, C) Mandibular neural crest. (D) Hyoid and mandibular neural crest. (E) Branchial and hyoid neural crest. (F) Trunk/vagal neural crest. e: eye; o- otic vesicle; nt: neural tube. Epidermal immunostaining is likely to be non-specific background.

Sox10 is normally expressed in all streams of migratory neural crest and in the otic vesicle (Fig. 5A). This neural crest pattern is reproduced in the Sox10-GFP stable transgenic embryos (Fig. 5B, C), showing clear GFP fluorescence in the cephalic neural crest streams at all migratory stages (Fig. 5D, E). The expression of Sox10 in the otic vesicle (Fig. 5A, D) is only partially recapitulated in this transgenic frog (Fig. 5B, E), suggesting that not all of the regulatory elements for the Sox10 gene are present in the 5.1 kb fragment. However, the expression of GFP in the neural crest is strong enough to allow direct visualization of GFP fluorescence and to perform time-lapse imaging of the migrating neural crest (Movie S1). Histological sections of Sox10-GFP stable transgenic *Xenopus laevis* embryos followed by immunostaining against GFP show that GFP is expressed in the mandibular (Fig. 6A-D, pink arrows), hyoid (Fig. 6A, D, E purple arrows), branchial (Fig. 6A,E, green arrows) and trunk (Fig. 6A,F) neural crest.

Supplementary material related to this article can be found online at doi:10.1016/j.ydbio.2018.02.020.

The following is the Supplementary material related to this article Movie 1.

Movie 1 Time lapse imaging of a Sox10-GFP stable transgenic embryo. Images were taken every 6 minutes over 12 hours using a Leica M205 FA Stereo fluorescence microscope.

These results show that this new Sox10-GFP stable transgenic *Xenopus laevis* could be used to study neural crest migration in amphibians. This new tool will allow researchers to perform live imaging time lapse microscopy and in addition, could be used to specifically label neural crest cells for FACS-sorting and purification for further molecular studies. The Sox10-GFP stable transgenic line is maintained at the European Xenopus Resource Centre (EXRC) and at the National Xenopus Resource at the Marine Biology Laboratory (MBL) where they are available upon request.

## 4 Conclusions

We have generated both Pax3-GFP and Sox10-GFP transgenic *Xenopus laevis* frog lines in which most of the endogenous embryonic expression of Pax3 and Sox10 are recapitulated. Pax3-GFP could be used for neural crest induction studies, whereas Sox10-GFP would be ideal to study neural crest migration. Thus, these two transgenic lines represent invaluable new tools to mechanistically study these two distinct processes of neural crest development.
